# Live cell imaging reveals marked variability in myoblast proliferation and fate

**DOI:** 10.1186/2044-5040-3-10

**Published:** 2013-05-02

**Authors:** Sean M Gross, Peter Rotwein

**Affiliations:** 1 Department of Biochemistry and Molecular Biology, Oregon Health & Science University, 3181 SW Sam Jackson Park Road, Portland, OR, 97239-3098, USA

**Keywords:** Live cell imaging, Single cell analysis, Cell death, Insulin-like growth factors

## Abstract

**Background:**

During the process of muscle regeneration, activated stem cells termed satellite cells proliferate, and then differentiate to form new myofibers that restore the injured area. Yet not all satellite cells contribute to muscle repair. Some continue to proliferate, others die, and others become quiescent and are available for regeneration following subsequent injury. The mechanisms that regulate the adoption of different cell fates in a muscle cell precursor population remain unclear.

**Methods:**

We have used live cell imaging and lineage tracing to study cell fate in the C2 myoblast line.

**Results:**

Analyzing the behavior of individual myoblasts revealed marked variability in both cell cycle duration and viability, but similarities between cells derived from the same parental lineage. As a consequence, lineage sizes and outcomes differed dramatically, and individual lineages made uneven contributions toward the terminally differentiated population. Thus, the cohort of myoblasts undergoing differentiation at the end of an experiment differed dramatically from the lineages present at the beginning. Treatment with IGF-I increased myoblast number by maintaining viability and by stimulating a fraction of cells to complete one additional cell cycle in differentiation medium, and as a consequence reduced the variability of the terminal population compared with controls.

**Conclusion:**

Our results reveal that heterogeneity of responses to external cues is an intrinsic property of cultured myoblasts that may be explained in part by parental lineage, and demonstrate the power of live cell imaging for understanding how muscle differentiation is regulated.

## Background

Muscle regeneration following injury occurs through stimulation of muscle stem cells, termed satellite cells [1]. Once activated, satellite cells proliferate to repopulate the injured area, and then exit the cell cycle to differentiate and eventually fuse to form new myofibers [1,2]. A similar series of steps occurs during muscle differentiation in culture. Yet, in both situations not all cells exposed to the same milieu have the same outcome. Some myoblasts continue to proliferate, others die, and another fraction becomes quiescent [3-6]. Because proliferation and death can occur simultaneously within a population, and can skew the fraction of cells that ultimately differentiate, it has been challenging to determine why some cells adopt one fate rather than another.

Muscle differentiation in culture has been studied primarily using endpoint assays that average cellular responses across the entire population. These assays require analyzing different cohorts of cells at different times and have inherently low temporal resolution. Furthermore, most endpoint assays assume homogeneity across the entire population. This assumption has been increasingly questioned by single cell measurements in other systems that find extensive variability within a population with regard to several critical parameters, including levels of gene or protein expression, responses to growth factor-activated signaling pathways, cell-cycle progression, and viability [7-11].

Live cell imaging resolves several limitations inherent in endpoint assays by allowing the same cells to be tracked with high temporal and spatial fidelity. This significantly improves the amount and quality of acquired data [12,13]. Furthermore, when combined with lineage tracing, live cell imaging can lead to insights regarding how cell fate decisions occur [8]. These approaches are especially important when identifying mechanisms controlling differentiation, in which a decision regarding the outcome of individual cells could be based on a niche signal, but could also be heritable or stochastic [14,15].

Here we have used live cell imaging and lineage tracing to assess both proliferation and the early phases of differentiation in the C2 muscle cell line. Our results reveal marked variability in both lineage size and fractional survival, but remarkable homogeneity within individual lineages in terms of cell fate and behavior. We also assessed the impact of IGF-I treatment, and found that although myoblast proliferation and survival increased, cell fate remained similar within lineages. These experiments suggest that myoblast fate is not stochastic, and provide an approach for discerning how various treatments might alter satellite cell behavior and function.

## Methods

### Materials

Fetal and newborn calf serum was purchased from Hyclone (Logan, UT, USA). Horse serum, goat serum, Dulbecco’s modified Eagle’s medium (DMEM), and PBS were from Life Technologies (Carlsbad, CA, USA). Porcine gelatin was from Sigma (St. Louis, MO, USA), Hoechst 33258 nuclear dye, from Polysciences (Warrington, PA, USA), and R3-IGF-I from GroPep (Adelaide, Australia). The primary antibody to troponin-T (CT3 from J. J-C. Lin) was purchased from the Developmental Studies Hybridoma Bank (Iowa City, IA, USA), and the secondary antibody, AlexaFluor 594-conjugated-goat anti-mouse IgG, was from Life Technologies. C2 myoblasts were obtained from Yaffe and Saxel [16], and HEK293FT cells were from Life Technologies. Other chemicals were reagent grade and were purchased from commercial suppliers.

### Development of a recombinant lentivirus expressing EGFP

A recombinant lentivirus was generated to express enhanced green fluorescent protein (EGFP) under control of the EF-1α promoter using as a base Addgene plasmid #12258 (Cambridge, MA, USA). Lentivirus was prepared in HEK293FT cells and purified as described [17,18]. Prior to use the virus was diluted in DMEM plus 2% fetal calf serum, and filtered through a 0.45 μM Gelman syringe filter (Pall Life Science, Ann Arbor, MI, USA).

### Cell culture

C2 myoblasts were grown and expanded on tissue culture plates coated with 0.2% gelatin in growth medium (DMEM, 10% heat-inactivated fetal calf serum, and 10% heat-inactivated newborn calf serum), as described [19,20]. For generation of EGFP-expressing C2 cells, myoblasts were transduced with the EGFP lentivirus as indicated [21]. Over 90% of cells expressed the recombinant protein, and EGFP expression persisted at comparable levels for more than five additional passages. EGFP-positive and control C2 myoblasts were grown separately and mixed at a 1:4 ratio prior to plating for live cell imaging. Using a mixed population of myoblasts at this ratio makes it possible to track labeled cells in dense populations, and is also amenable to automated tracking, which was not possible from bright field images. For live cell imaging experiments, cells were plated on 6-well plates and then immediately placed in the IncuCyte FLR (Essen Biosciences, Ann Arbor, MI, USA), a microscopy system located inside a standard tissue culture incubator. The incubator was maintained at 37°C in humidified air with 5% CO_2_. Bright field and EGFP images were acquired at 10× magnification from four locations per well at 15-min intervals in order to accurately and completely track all labeled cells. The four locations, which were predefined by the imaging system and consistent across all experiments, were arranged as a square, with each point equidistant from the midpoint of the well. After 24 h in growth medium cells were washed with PBS, and differentiation medium (DM, DMEM with 2% horse serum) was added. For selected wells, R3-IGF-I [2 nM] was added with DM.

### Image analysis

To quantify cell number a module was created using the Cell Profiler software program [22] that loaded EGFP images as a batch, converted images to grayscale, and performed an illumination correction. EGFP-positive cells in each field were identified by applying a background adaptive threshold, which separates primary objects from the background by setting a threshold at twice the value of the mode of the histogram for pixel intensity. Objects identified by the algorithm that were below 15 microns in diameter were discarded from the automated count. Cell confluence was calculated for each field using an IncuCyte algorithm, which quantifies the relative cell area in each bright field image.

**Figure 1:**
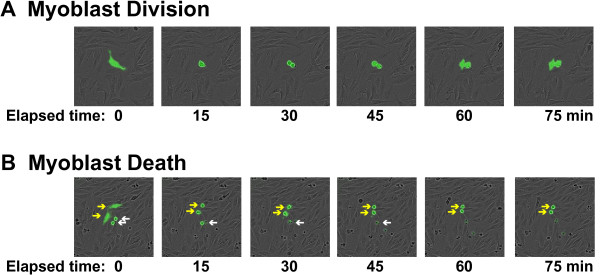
**Visualizing myoblast division and death.** (**A**) Time lapse images (EGFP and bright field) at 15 min increments of an EGFP-labeled myoblast undergoing mitosis. (**B**) Time lapse images (EGFP and bright field) at 15 min increments of myoblast death. The arrows point to EGFP-labeled myoblasts. The white arrows indicate cells that began to die prior to the first frame, and illustrate loss of EGFP fluorescence and detachment over time of observation. The yellow arrows mark cells that condensed and underwent death during the period of observation. Loss of EGFP fluorescence and detachment occurred during the subsequent 60 min (not shown).

For our studies we have defined a founder as a cell present at the time of plating, and a lineage as all of the progeny of a founder cell. To follow lineages, founder cells and all progeny were manually tracked using registered EGFP images starting from the first image obtained. Cells around the border of each field were excluded from analysis as most tended to exit the viewing area over the course of an experiment. Both cell division and death were readily identified and quantified (see Figure 1). Cell death was easily detected, as it ultimately culminated in cell lysis, and was preceded by condensation, blebbing, and loss of EGFP fluorescence. Additionally, an advantage of live cell imaging is that subsequent tracking of same area of the field could confirm that death occurred.

### Immunocytochemistry

To analyze muscle differentiation, plates that had been imaged for 90 h (24 h in growth medium plus 66 h in DM) were washed with PBS, fixed with paraformaldehyde for 10 min, and washed again with PBS followed by a 90-s treatment with 50% acetone - 50% methanol, as described [19,20]. After three additional PBS washes wells were incubated with 0.25% goat serum in PBS for ≥2 h to block non-specific antibody binding, followed by incubation overnight at 4°C with troponin-T primary antibody (1:100 dilution), washing with PBS, and incubation for 90 min at 20°C with AlexaFluor 594-conjugated-goat anti-mouse IgG (1:3,000 dilution) and Hoescht nuclear dye. Cells were visualized with a Nikon Eclipse Ti-U inverted microscope and a Nikon DS-Qi1Mc camera using the NIS elements 3.1 software.

### Statistical analysis

To assess observed cell viability data, we first calculated the number of living and dead cells among sibling pairs. Cells that failed to divide, or that had a sibling that underwent a second division, were not entered into this analysis since they had no comparable sibling. This excluded 16 cells, which had a percent survival of 25%. Assuming random death between pairs of cells, we calculated the expected number of pairs composed of two living myoblasts, a living and a dead cell, or two dead cells. Expected frequencies were then compared to observed data using a χ-squared test with two degrees of freedom. To test for a relationship between a variable in sibling pairs (cell cycle duration or time to death), we calculated the Pearson correlation coefficient. To test for a correlation between random cells, we randomized the pairings and performed the same test. Results were considered statistically significant when *P* ≤0.01.

## Results

### Defining myoblast dynamics by live cell imaging

**Figure 2:**
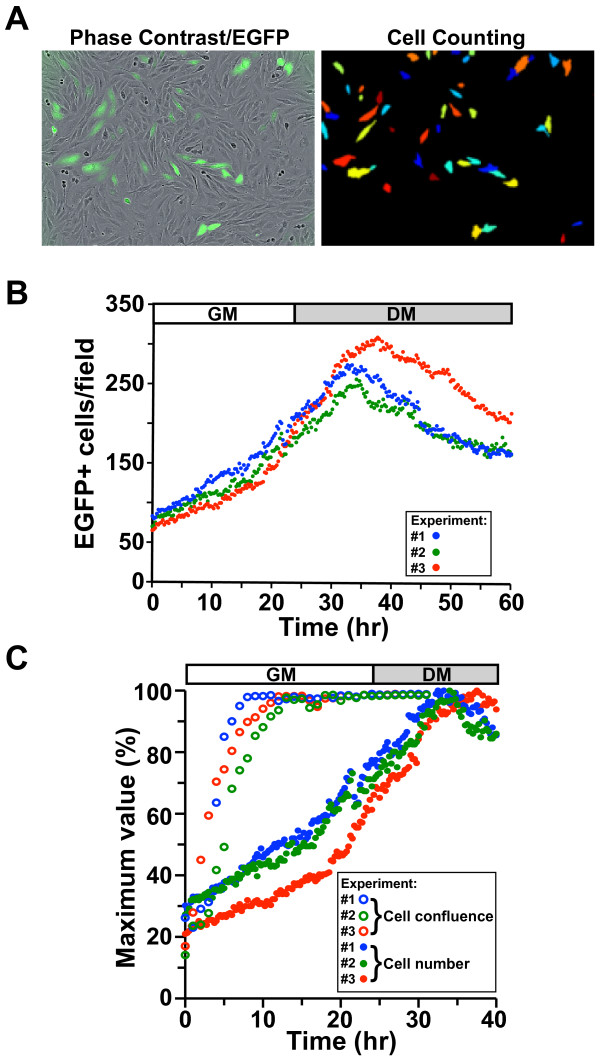
**Defining myoblast dynamics by live cell imaging.** C2 myoblasts were mixed at a 1:4 ratio with C2 cells stably infected with an EGFP gene under control of the EF-1α promoter, and the EGFP-expressing myoblasts were tracked at 15-min intervals using an automated cell counting algorithm. See ‘Methods’ for additional details. (**A**) Phase contrast image of EGFP-positive myoblasts (left) and the corresponding image of the same microscopic field with EGFP-expressing cells identified by an automated cell counting algorithm. (**B**) Cell number as a function of time in culture for three independent experiments (blue, green, and red). Each dot represents a single measurement. (**C**) Percentage of maximum cell number (closed dots) and percent confluence (open dots) as a function of time in culture for three independent experiments (blue, green, and red).

We employed live cell imaging to track myoblast proliferation and monitor survival during a differentiation time course. To study myoblast dynamics, we plated a mixture of unmarked myoblasts with myoblasts expressing EGFP under control of the constitutively active EF-1α promoter, and tracked EGFP-positive cells every 15 min using an automated cell counting algorithm (Figure 2A). We found that a mixed population was necessary for accurate tracking once the cells reached confluence. We observed a progressive increase in cell number with an average doubling time of 17.6 h during the initial 24 h of incubation (Figure 2B). After 24 h, high serum growth medium was replaced with low serum differentiation medium (DM). Following addition of DM, cell number continued to increase, leading to a peak in myoblast number between 8 and 14 h after medium was changed. Cell number then progressively declined, but began to stabilize by the end of the recording period after 36 h in DM (Figure 2B). When myoblasts were plated at similar densities these patterns were consistent across multiple locations in a single well and across independent experiments (Figure 2B and Additional file 1: Figure S1A, B), but varied in degree and timing when cells were plated at higher or lower densities (Additional file 1: Figure S1C).

Tracking cells beyond 60 h revealed that EGFP-positive myoblasts fused with both EGFP-expressing and non-labeled cells to form multinucleated myotubes (Additional file 2: Figure S2). These results were confirmed by identifying troponin-expressing cells by immunocytochemistry (Additional file 2: Figure S2). Thus, neither EGFP expression nor live cell imaging compromised muscle differentiation.

Since confluence is frequently used to establish when DM is added, we tracked confluence and compared it to measures of cell number. Immediately upon plating, confluence was approximately 20% and cell number was approximately 25% of its maximum value (Figure 2C). Cells soon spread out and began to divide so that by 10 h in culture when the EGFP-positive myoblast number per field was approximately 30% to 40% of maximal, confluence had reached approximately 85% to 95% (Figure 2C). By 24 h in growth medium when the cell number was approximately 70% to 80% of maximal, confluence was approximately 99%, and it remained constant despite a further rise in myoblast number (Figure 2C). Thus, confluence and cell number are poorly correlated.

### Defining myoblast population kinetics

Our automated counting algorithm measured changes in cell number, but was unable to quantify individual instances of cell death or division. In order to quantify death and division, we manually tracked myoblasts and their progeny over a 60-h incubation period. Both cell division and death could be readily detected and monitored (Figure 1 and Additional files 3 and 4: Movie). During cell division, cells condensed into a circular shape, which was followed by mitosis and emergence of two progeny (Figure 1A). Cell death was detected by shrinkage, blebbing, lysis, and the ultimate loss of EGFP fluorescence (Figure 1B). Comparing manual and automated measures of the total cell number revealed similar kinetics, thus validating the automated cell counting algorithm (Additional file 1: Figure S1A, B).

**Figure 3:**
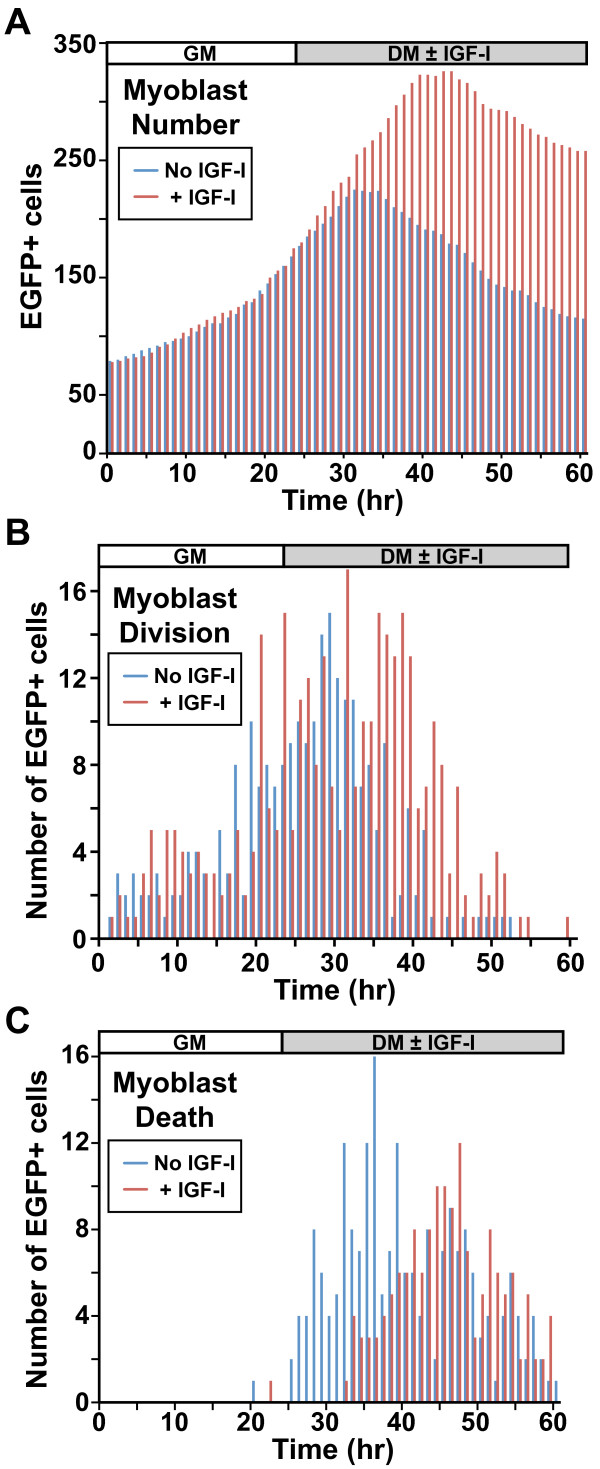
**Defining myoblast population kinetics by live cell imaging.** Individual EGFP-expressing myoblasts were studied as in Figure 2 using manual counting. Differentiation medium (DM) was added ± IGF-I (R3-IGF-I [2 nM]), as indicated. (**A**) Effect of IGF-I on total cell number. (**B**) Effect of IGF-I on the frequency of cell division (DM 234 divisions; IGF-I 344 divisions). (**C**) Effect of IGF-I on myoblast death (DM 208 deaths; IGF-I 154 deaths). For A to C, blue depicts control cells and red, myoblasts incubated with IGF-I. For B and C, the number of cells exhibiting a specific trait at a given time is plotted on the y-axis.

Cell tracking revealed that myoblast proliferation continued well after DM was added (Figure 3A, B, Additional file 5: Figure S3). Cell death was largely absent during the 24 h in GM, but was extensive after addition of DM so that cell division and death were occurring simultaneously (Figure 3C, Additional file 5: Figure S3). Addition of IGF-I ([2 nM] R3-IGF-I) with DM led to a rise in the maximal myoblast number over controls (Figure 3A). This was a consequence of an increase in cell division and a reduction in myoblast death (Figure 3B, C).

### Myoblast lineage analysis

**Figure 4:**
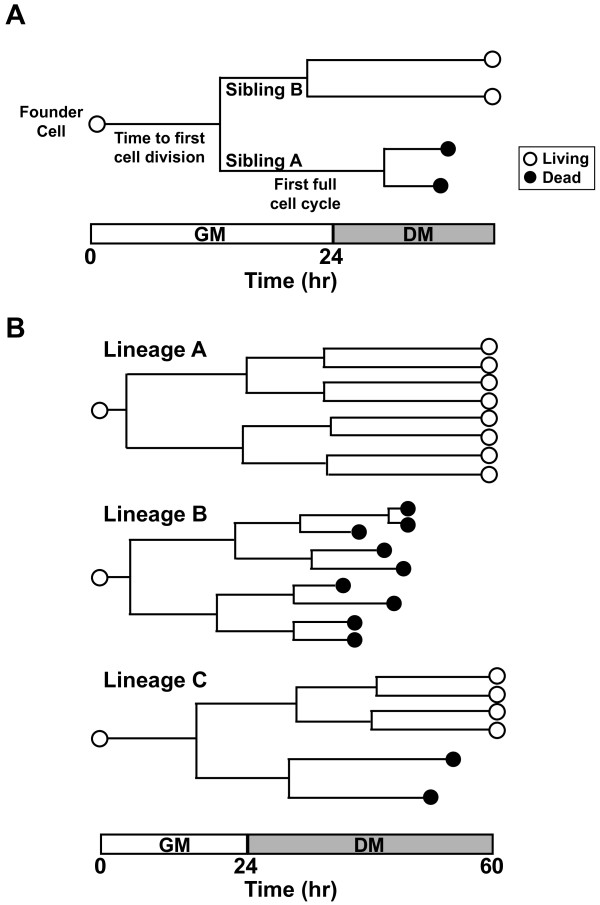
**Studying the fate of individual myoblasts.** (**A**) Schematic of a hypothetical myoblast lineage tree, with different features indicated on the timeline. (**B**) Examples of three actual cell lineages with progeny and fates of individual cells indicated.

To assess myoblast fate, we tracked 79 founder cells and their progeny starting from the time of plating, and measured multiple kinetic parameters (Figure 4A). For the purpose of our studies, we define lineage as all the progeny of a single cell, and fate as a specific outcome (for example, survival, death, differentiation). For each lineage, we recorded the duration from the start of imaging until a founder cell divided, labeled as the time to the first cell division (Figure 4A). This initial division produced two cells, sibling A and sibling B. The time from the first cell division to the division of each sibling was recorded as the first full cell cycle (Figure 4A). Data from three lineages that varied in outcomes are depicted in Figure 4B.

**Figure 5:**
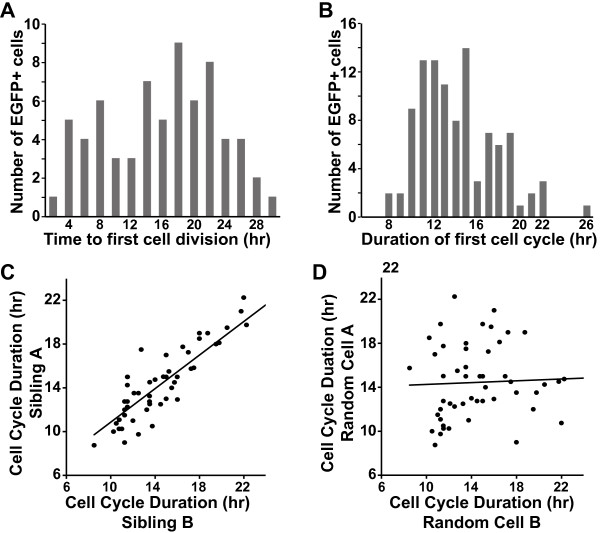
**The cell-cycle duration of individual C2 myoblasts is heterogeneous.** Individual EGFP-expressing myoblasts were monitored at 15-min intervals as in Figures 1 and 2. (**A**) Asynchronous entry of myoblasts into the cell cycle, as measured by the time from plating until the first cell division. The number of myoblasts undergoing mitosis at a given time is plotted on the y-axis. (**B**) Frequency distribution of the duration of the first full cell cycle. The number of myoblasts exhibiting a given cell cycle duration is plotted on the y-axis. (**C**) Close correlation (0.85, Pearson correlation coefficient) of cell cycle duration between siblings (progeny of the same cell division; *P* = 1.834e^-13^, t = 11.14, degrees of freedom (DF) = 49). (**D**) No correlation was observed for randomly paired cells (-0.07; *P* = 0.631, t = -0.4834, DF = 49)).

By tracking the time from plating until the first cell division, we found a relatively broad distribution that ranged from 2 to 30 h (Figure 5A). This illustrated that the start of cell division was asynchronous in the population. We next tracked cell cycle duration using the first full cell cycle following the division of each founder cell. This varied across the population from 8 to 26 h with a mean of 14.2 h (Figure 5B). The mean cell cycle duration was shorter than the population doubling time, due in part to eight of 79 founder myoblasts that failed to divide over the entire 60-h time course.

Despite the range of cell cycle durations in the population, there was a remarkably close correlation between siblings (Figure 5C). This relationship was not detected between cells paired randomly (Figure 5D). Between siblings the Pearson correlation coefficient for cell cycle duration was 0.85 (*P* = 1.834e^-13^), but between random pairs of cells it was -0.07 (*P* = 0.631).

**Figure 6:**
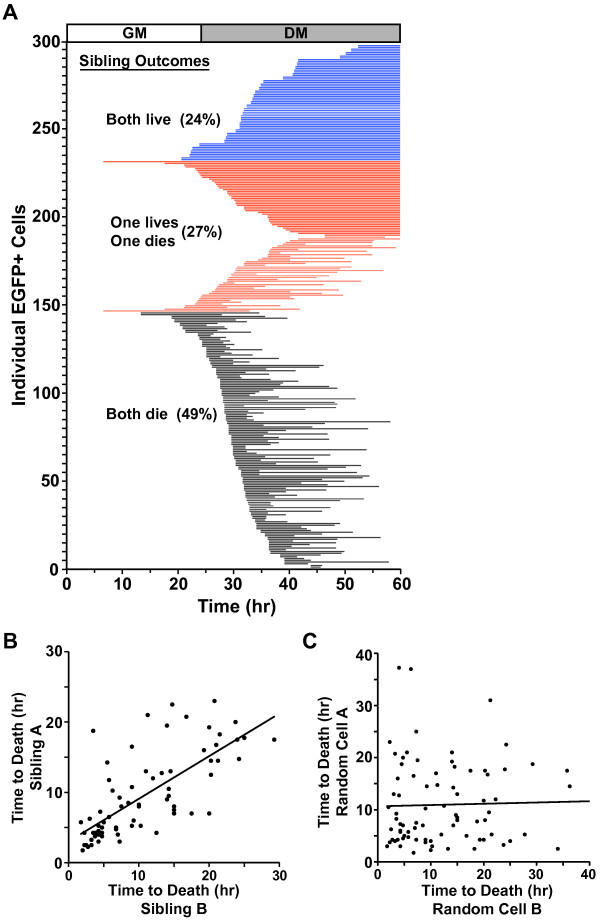
**Concordance of myoblast fate.** Individual EGFP-expressing myoblasts were analyzed at 15-min intervals as in Figures 1 and 2. (**A**) The line plot shows the fate of each myoblast (*n* = 298). Each horizontal line indicates a survival timeline for a single myoblast with the left end representing the time after the last cell division (= starting point), and the right end indicating either the time of death or survival to 36 h in DM. Concordance or discordance of outcomes between siblings is indicated (black and blue lines reflect concordance, red discordance). The number of identical fates between siblings was significantly larger than expected by chance (*χ*^2^ = 21.064, DF = 2, two-tailed *P* <0.0001). (**B**, **C**) Correlation of time of cell death for siblings (Pearson correlation coefficient between sibling cells was 0.7196 (*P* <2.247e^-12^, t = 8.733, DF = 71) and between randomly paired cells was 0.1163 (*P* <0.3271, t = 0.9868, DF = 71)).

We next assessed cell viability, since it has been shown that a significant fraction of myoblasts undergo apoptotic death during incubation in DM [23-27]. For this analysis, we compared the survival of 149 sibling pairs (298 total cells). As depicted in Figure 6A, over 60% of cells died in DM. When survival and death were assessed on the basis of parentage, we found that 73% of siblings had concordant fates, with 49% both dying and 24% both living, and 27% were discordant, with one myoblast living and the other dying (Figure 6A). The number of shared fates between siblings was significantly larger than expected if survival occurred solely by chance (values expected if cell death is random: 40.3% both die, 13.3% both live, 46.4% discordant (*P* <0.0001)).

Similarly, even though incubation with IGF-I reduced the percentage of cells that died (Figure 3), concordance among siblings was 75% (50% both living and 25% both dying, Additional file 6: Figure S4). This bias toward concordant sibling fates was nearly identical to that observed in cells incubated with DM alone (Figure 6A), despite the percentages of both myoblasts living and both dying being reversed (*P* <0.0001). These results indicate that survival was not purely stochastic, but instead was biased by parental lineage.

When the time from last division to death was tracked between concordant siblings (Figure 6B), we found a close correlation similar to that seen with cell cycle duration, further reinforcing the importance of parental lineage. The Pearson correlation coefficient for time to death between siblings was 0.72 (*P* = 2.247e^-12^), while by contrast between random cells the value was 0.12 (*P* = 0.3271) (Figure 6C).

### Heterogeneity among myoblast lineages

**Figure 7:**
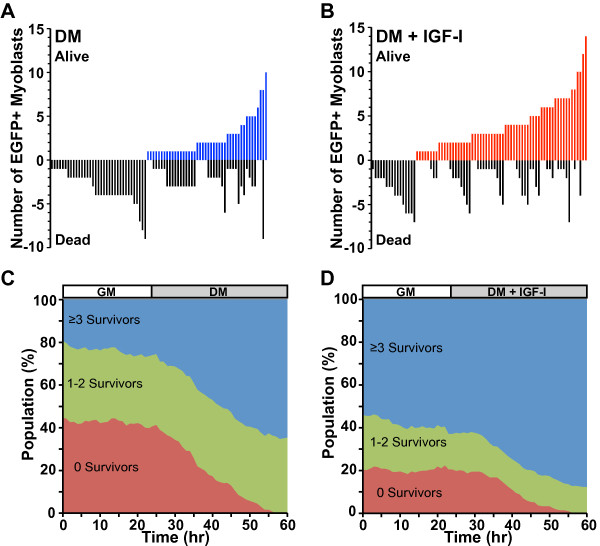
**Myoblast lineages are heterogeneous.** EGFP-expressing myoblasts were studied as in Figure 1. Differentiation medium (DM) was added ± IGF-I (R3-IGF-I (2 nM)), as indicated. (**A**, **B**) Line plots showing the number of cells derived from each lineage and the outcome (alive or dead) tracked on the y-axis. (**C**, **D**). Variation in outcomes of progeny for individual founder myoblasts leads to a shift in the population. The population number was normalized across time. Red, founder cells and their progeny with zero surviving myoblasts; green, founders with 1 to 2 survivors; blue, lineages with ≥3 survivors.

We next sought to analyze how concordance between siblings altered lineage outcomes during muscle differentiation. We found that lineage sizes were unequal as a consequence of variable rates of cell division and survival. A fraction of lineages failed to divide, another fraction underwent fewer than two cell divisions, and another had multiple divisions (Figure 7). Myoblast survival also was heterogeneous, as some lineages of similar size maintained 100% viability, others underwent 100% death, and others had mixed outcomes (Figure 7A). Incubation of myoblasts in DM with IGF-I led to a higher fraction of lineages with 100% survival, but IGF-I was not able to rescue all lineages since 16 (approximately 20%) still underwent complete death (Figure 7B). Thus, myoblast lineage size and viability were variable.

To assess how heterogeneity in lineage size or survival might be reflected in the total population after a differentiation time course, we plotted the number of living myoblasts in each lineage over time, grouping lineages according to outcome. We found that the population was evenly represented by each of the founder cell lineages during incubation in growth medium, but not after addition of DM. One group of myoblasts, comprising approximately 40% of the initial population (Figure 7C, green tracing), maintained a similar representation for the entire culture period, while another equivalently sized group of founders failed to have one cell survive after incubation in DM (Figure 7C, red). In contrast, a third group significantly expanded from approximately 20% of the initial population to approximately 60% of the final cohort (Figure 7C, red). Thus, the overall population at the end of the experiment differed substantially from the population at the start.

In myoblasts incubated in DM plus IGF-I the relative number of lineages in each group was different. IGF-I treatment resulted in only 20% of founders not being represented in the final population, and 57% of founders comprised 85% of the final group (Figure 7D). Thus, addition of IGF-I in DM maintained the myoblast lineage distribution so that it more closely resembled the population at the start.

## Discussion

Here we have used live cell imaging and lineage tracing to address the dynamics of muscle cell proliferation and survival in the C2 myoblast cell line. We find a wide variation in the rate and extent of both proliferation and viability of myoblasts derived from different parental cells, but concordant behavior in cells arising from the same parents. As a consequence, the population of myoblasts undergoing differentiation varied substantially from the cells present at the start of an experiment. Addition of IGF-I to DM reduced population heterogeneity primarily by sustaining myoblast viability, and thus increased the number and sizes of surviving lineages. As a result, the terminal population more closely resembled the cohort of myoblasts at the start than it did in untreated cells. Our observations reveal that under standard treatment protocols extensive heterogeneity is an intrinsic property of cultured myoblasts, and that an effect of IGF-I is to decrease this variability.

### Myoblast population features

We found that cell cycle durations were heterogeneous across the population and that cell division continued after DM was added. The average cell cycle duration of the population in growth medium was 14.2 h. This value matches doubling times of C2 myoblasts obtained by other approaches, including labeling of DNA synthesis and direct cell counting [23], but was easier to acquire and potentially more accurate, since individual myoblasts were tracked rather than averaging multiple time points from different groups of cells. Despite agreement of our data with previous observations, we found that the cell cycle duration of individual myoblasts varied extensively (range, 8–26 h), and that approximately 10% of the founder cells failed to divide once over the entire 60-h tracking period. Remarkably, these observations are similar to results obtained with satellite cells derived from dissociated single muscle fibers [28], where both the onset of proliferation and individual cell cycle durations varied in the population (range, 5.1-17.8 h), and approximately 16% of cells failed to divide once [28].

By assessing myoblast lineage, we observed that there was a close correlation between the cell cycle durations of siblings (Figure 5C), in remarkable agreement with an observation noted more than 25 years ago in primary quail myoblasts [29]. These similarities between cells of shared parentage in both primary myoblasts and a muscle cell line point to the potential importance of heritability and the immediate environment in regulating cell fate.

Myoblast differentiation requires exit from the cell cycle in G1 [30-32]. Since our data showed that cell division continued well after addition of DM in a fraction of cells, this indicates that the onset of muscle differentiation is heterogeneous. Furthermore, since IGF-I treatment prolonged the time of cell division, it is likely to increase the duration over which cells exit the cell cycle (Figure 3B). This problem of variability is further compounded by methods that rely on confluence to mark the time when DM should be added [33,34], since confluence is relatively difficult to visually quantify, and as seen here, small changes in confluence can equate to large differences in cell numbers (Figure 2B).

It is well known that a fraction of cultured myoblasts succumb to apoptotic cell death during incubation in DM [23-25,35]. Similarly, it has been reported that in response to muscle injury *in vivo*, satellite cell proliferation is followed by a period of satellite cell death [6]. We found that the net decline in cell number following 36 h in DM was approximately 35%. This is in line with the 20% to 30% value previously reported with endpoint methods, including TUNEL assays, cell counting, and ‘live-dead’ staining [25,35]. Yet, by tracking both the division and death of individual cells, we found that over 60% of the population died during 36 h in DM, with the majority of death occurring during the initial 15 h (Figure 3C). This value differed substantially from cell number calculations because of concurrent proliferation of other EGFP-labeled myoblasts during incubation in DM. Thus, we suggest that traditional apoptosis assays underestimate the extent of myoblast death by failing to account for ongoing proliferation of other cells in the culture.

By identifying and tracking individual myoblasts and their offspring, we found that cell death was not random. Rather, siblings were biased toward adopting concordant fates after incubation in DM. The tendency toward common outcomes was maintained even in cells exposed to IGF-I, despite enhanced myoblast survival from IGF-I treatment (Figure 3C, Additional file 4: Figure S4). The mechanisms responsible for these biases are unclear, but could arise from genetic or epigenetic differences, or from environmental influences. We suspect that a fraction of the bias may be explained by similar levels or activity of the IGF-I - PI3-kinase - Akt signaling pathway between related cells, since exposure to IGF-I reduced myoblast death, but maintained concordant fates between siblings. It is therefore possible that cells of shared parentage inherit similar amounts of signaling components, and/or share epigenetic or genetic alterations that affect regulation of this pathway. This is consistent with observations that cell siblings adopt concordant fates in response to apoptosis-inducing agents because of a common inheritance of proteins from their mother [7,8]. Alternatively, as siblings share a similar microenvironment, we cannot exclude the possibility that paracrine factors also contribute to the regulation of cell survival.

The main impact of cell death not being random was a dramatic change in the composition of the myoblast population by the end of the culture period. This was not apparent during the initial 24 h of incubation in growth medium because myoblast viability was complete and most of the cells underwent at least one cell division (Figure 5). Variability arose, however, during the subsequent 36 h in DM, as distinct subpopulations developed rapidly from heterogeneous cell division coupled with variable survival. This led to substantial differences in the contributions of different lineages to the final myoblast population (Figure 6A, 7, Additional file 6: Figure S4). Our results suggest that measurements that average cellular characteristics during a differentiation time course, such as immunoblots or gene expression assays, can obscure the properties of subpopulations.

### Impact of IGF-I on myoblast proliferation, survival, and differentiation

IGF-I exerts potentially contradictory effects on muscle cells, including promoting both proliferation and differentiation [33]. Our observations suggest one resolution to this problem. Analysis of the onset of the last division revealed that IGF-I led to an average delay of approximately 5 h compared with untreated controls (Figure 3B, Additional file 6: Figure S4). As this delay did not lead to more than one additional cell division, our interpretation is that the main action of IGF-I is to maintain myoblast survival so that otherwise vulnerable cells are able to complete a single final round of replication. These effects of IGF-I complicate comparisons with untreated cells, as both fractional myoblast survival and the starting points for differentiation are different. Future applications of reporters for different aspects of differentiation are needed to improve our understanding of the kinetics and regulation of muscle differentiation by separating out these confounding factors.

### Satellite cell fate and muscle regeneration

An important question in skeletal muscle biology is how satellite cell fate is regulated during muscle regeneration. Following injury, satellite cells must divide sufficiently to insure adequate numbers of differentiating myoblasts for immediate muscle repair, but also must maintain a reserve population for regeneration after subsequent injury [4,6,36]. Thus, multiple cell fate decisions are necessary to ensure adequate current and future muscle repair. Live cell imaging has begun to be applied to this question, and has revealed the importance of asymmetric and symmetric satellite cell divisions [14,37,38]. Other studies have shown that the satellite cell population is very heterogeneous, not only in terms of its behavior in response to proliferative or migratory cues [28,39], but also with respect to cell of origin [36], and surface protein expression [6,40]. Thus both environmental and genetic factors potentially play influential regulatory roles in muscle regeneration through effects on satellite cells. Elucidating how this heterogeneity impacts the decisions that lead to satellite cell differentiation will be critical to understanding the dynamics of muscle regeneration and how under certain circumstances such as aging this process can go awry.

## Conclusions

We have used live cell imaging and lineage tracing to assess both proliferation and the early events of differentiation in C2 myoblasts. Our results reveal marked variability in lineage size and fractional survival, but remarkable homogeneity within individual lineages in terms of cell fate. Treatment with IGF-I increased myoblast number by maintaining viability and by stimulating a fraction of cells to complete one additional cell cycle in DM, and as a consequence reduced the variability of the terminal population compared with controls. Our results reveal that heterogeneity is an intrinsic property of cultured myoblasts, and demonstrate the power of live cell imaging to provide insights into the regulation of muscle differentiation.

## Abbreviations

DM: Differentiation medium; DMEM: Dulbecco’s modified Eagle’s medium; EGFP: Enhanced green fluorescent protein; GM: Growth medium; IGF-I: Insulin like growth factor-I; PBS: Phosphate buffered saline.

## Competing interests

The authors declare that they have no competing interests.

## Authors’ contributions

SMG and PR conceived of the research; SMG performed the research; SMG and PR analyzed the research and wrote the manuscript. Both authors read and approved the final manuscript.

## Supplementary Material

Additional file 1: Figure S1 Characterizing myoblasts by live cell imaging. C2 cells were mixed at a 1:4 ratio with C2 myoblasts stably infected with an EGFP gene under control of the EF-1α promoter. The EGFP-expressing myoblasts were tracked at 15-min intervals. **(A)** Concordance between results of manual and automated cell counting. Cells were incubated for 60 h, with DM ± IGF-I (R3-IGF-I [2 nM]) being added for the last 36 h (red traces). Solid lines represent manual tracking of lineages and dots represent automated counting. **(B)** Reproducibility of automated cell counting. Four wells were plated with an identical number of cells, and were incubated for 60 h, with DM ± IGF-I being added for the last 36 h. **(C)** Effects of plating density on myoblast dynamics. Cells were plated at varying concentrations, and EGFP-positive cells were identified by automated counting at 15-min intervals for 60 h. Click here for file

Additional file 2: Figure S2 EGFP-expressing myoblasts undergo differentiation. Confluent myoblasts were incubated in DM for 66 h. **(A)** Live cell images of EGFP fluorescence were captured (10× magnification). **(B)** Differentiating myoblasts were fixed and stained with antibodies to troponin-T (red), and nuclei were stained with Hoescht dye (blue, 100× magnification). Click here for file

Additional file 3: Movie 1 Live cell imaging of C2 myoblasts. Live cell imaging of C2 myoblasts for 60 h (24 h in growth medium, 36 h in DM). Fluorescent images were captured every 15 min. Click here for file

Additional file 4: Movie 1 Live cell imaging of C2 myoblasts with manual tracking overlay. Live cell imaging of C2 myoblasts for 60 h (24 h in growth medium, 36 h in DM). Fluorescent images were captured every 15 min. Click here for file

Additional file 5: Figure S3 Reproducibility of myoblast dynamics by live cell imaging. Individual EGFP-expressing myoblasts were manually tracked at 15-min intervals in three independent experiments, as in Figure 3. Left panels: cell number measured as a function of time in culture. Center panels: frequency of cell division analyzed as a function of time in culture. Right panels: frequency of myoblast death recorded as a function of time in culture. Click here for file

Additional file 6: Figure S4 IGF-I promotes myoblast proliferation and enhances viability. Individual EGFP-expressing myoblasts were analyzed at 15-min intervals as in Figures 3 and 6. The line plot shows the fate of each myoblast (*n* = 372). Each horizontal line indicates a survival timeline for a single myoblast with the left end representing the time after the last cell division (= starting point), and the right end indicating either the time of death or survival to 36 h in DM. Concordance or discordance of outcomes is indicated (black and blue lines reflect concordance, red discordance). The number of identical fates between siblings was significantly larger than expected by chance (*χ*^2^ = 45.581, DF = 2, two-tailed *P* <0.0001). Click here for file
