# Supplementary figures and images for: Live cell imaging reveals marked variability in myoblast proliferation and fate
Source: Skelet Muscle. 2013 May 2;3:10. doi: 10.1186/2044-5040-3-10 (PMC3712004; doi:10.1186/2044-5040-3-10)

# Supplemental Fig 1

## A Manual vs. Automated Cell Counting

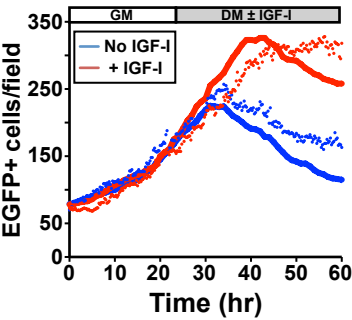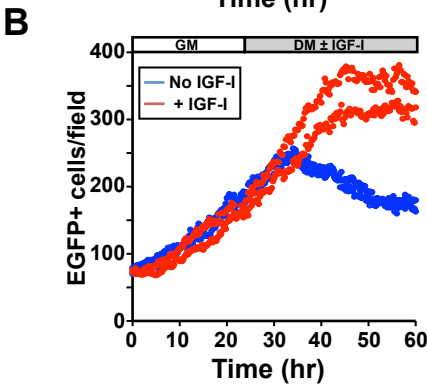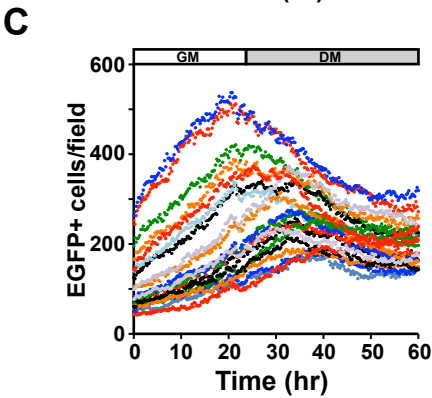

Supplement: Additional file 1: Figure S1 — Characterizing myoblasts by live cell imaging. C2 cells were mixed at a 1:4 ratio with C2 myoblasts stably infected with an EGFP gene under control of the EF-1α promoter. The EGFP-expressing myoblasts were tracked at 15-min intervals. (A) Concordance between results of manual and automated cell counting. Cells were incubated for 60 h, with DM ± IGF-I (R3-IGF-I [2 nM]) being added for the last 36 h (red traces). Solid lines represent manual tracking of lineages and dots represent automated counting. (B) Reproducibility of automated cell counting. Four wells were plated with an identical number of cells, and were incubated for 60 h, with DM ± IGF-I being added for the last 36 h. (C) Effects of plating density on myoblast dynamics. Cells were plated at varying concentrations, and EGFP-positive cells were identified by automated counting at 15-min intervals for 60 h. [file 2044-5040-3-10-S1.pdf]

Supplemental Fig 2

A

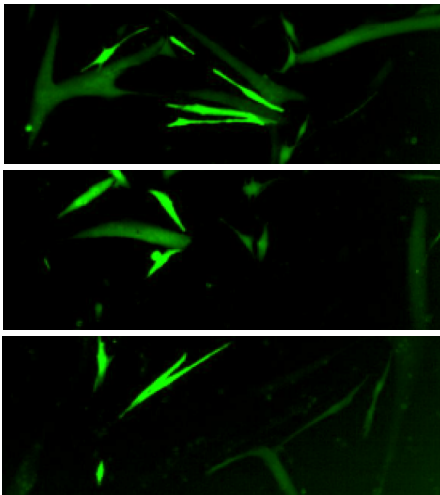

B

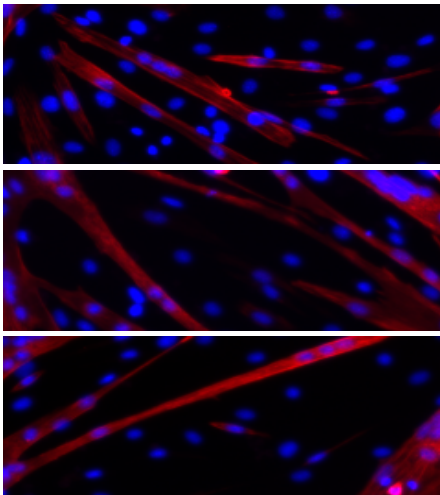

Supplement: Additional file 2: Figure S2 — EGFP-expressing myoblasts undergo differentiation. Confluent myoblasts were incubated in DM for 66 h. (A) Live cell images of EGFP fluorescence were captured (10× magnification). (B) Differentiating myoblasts were fixed and stained with antibodies to troponin-T (red), and nuclei were stained with Hoescht dye (blue, 100× magnification). [file 2044-5040-3-10-S2.pdf]

Supplemental Fig 3

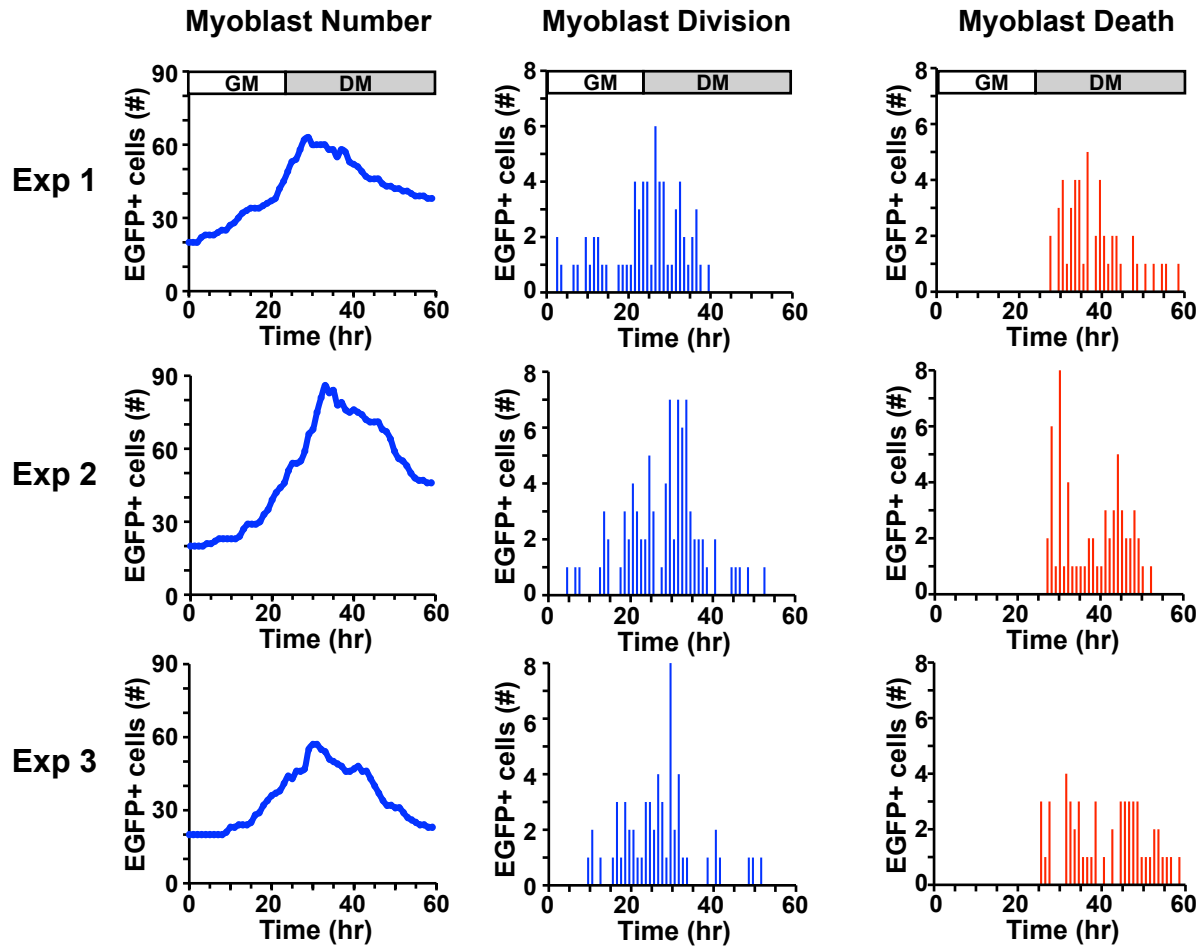

Supplement: Additional file 5: Figure S3 — Reproducibility of myoblast dynamics by live cell imaging. Individual EGFP-expressing myoblasts were manually tracked at 15-min intervals in three independent experiments, as in Figure 3. Left panels: cell number measured as a function of time in culture. Center panels: frequency of cell division analyzed as a function of time in culture. Right panels: frequency of myoblast death recorded as a function of time in culture. [file 2044-5040-3-10-S5.pdf]

Supplemental Fig 4

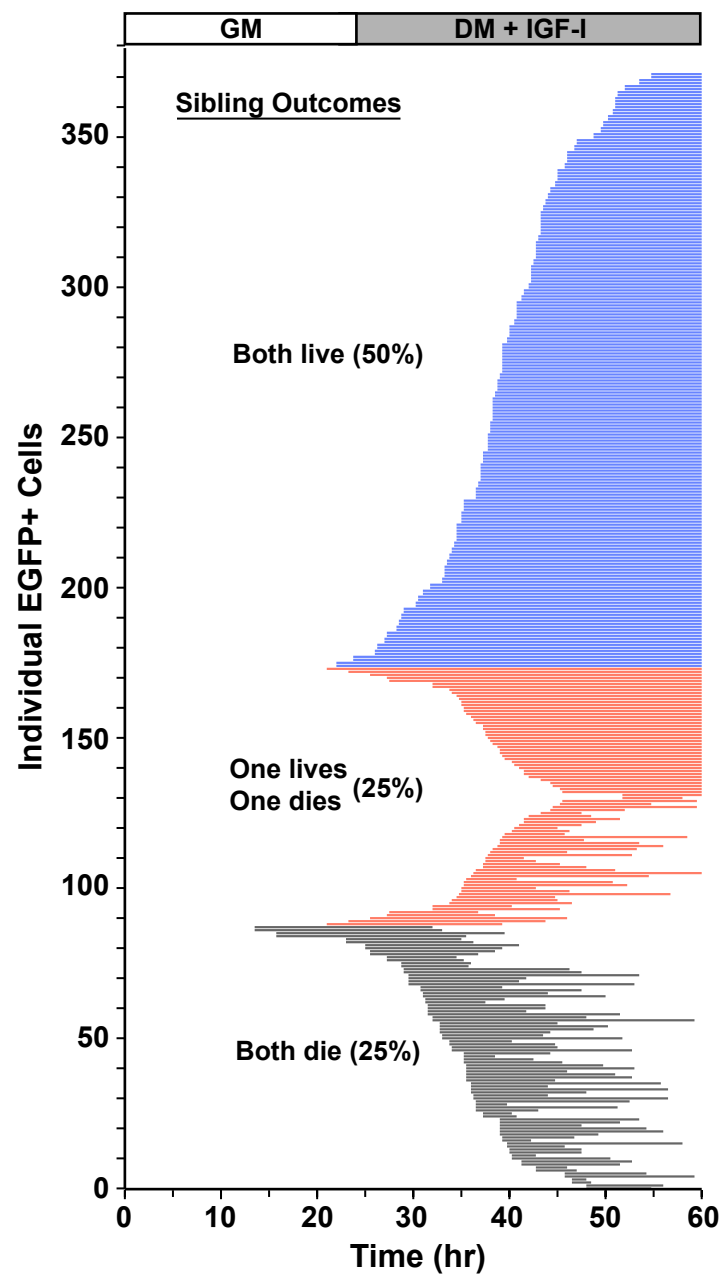

Supplement: Additional file 6: Figure S4 — IGF-I promotes myoblast proliferation and enhances viability. Individual EGFP-expressing myoblasts were analyzed at 15-min intervals as in Figures 3 and 6. The line plot shows the fate of each myoblast (n = 372). Each horizontal line indicates a survival timeline for a single myoblast with the left end representing the time after the last cell division (= starting point), and the right end indicating either the time of death or survival to 36 h in DM. Concordance or discordance of outcomes is indicated (black and blue lines reflect concordance, red discordance). The number of identical fates between siblings was significantly larger than expected by chance (χ2 = 45.581, DF = 2, two-tailed P <0.0001). [file 2044-5040-3-10-S6.pdf]
